# Consumption of psychoactive substances in prison: Between initiation and improvement, what trajectories occur after incarceration? COSMOS study data

**DOI:** 10.1371/journal.pone.0225189

**Published:** 2019-12-04

**Authors:** Morgane Rousselet, Marylène Guerlais, Pascal Caillet, Bertrand Le Geay, Damien Mauillon, Patrick Serre, Pierre-Yves Chameau, Yves Bleher, Serge Mounsande, Pascale Jolliet, Caroline Victorri-Vigneau

**Affiliations:** 1 Centre for Evaluation and Information on Pharmacodependence, Clinical Pharmacology Department, Nantes University Hospital, France; 2 INSERM U1246 SPHERE “methodS in Patient-centered outcomes and HEalth ResEarch”, Nantes and Tours University, Nantes, France; 3 Addictology and Psychiatry Department, University Hospital, Nantes, France; 4 Department of Prison Psychiatry, Nantes University Hospital, France; 5 Medical department of prison, Angers University Hospital, France; 6 Medical department of prison, Le Mans HospitalFrance; 7 Medical department of prison, Laval Hospital, France; 8 Medical department of prison, La Roche sur Yon Departemental Hospital, Boulevard Stéphane Moreau, France; 9 Medical department of prison, Fontenay-Le-Comte Hospital, France; University of Kansas, UNITED STATES

## Abstract

**Background:**

Few studies have examined the consumption trajectories of inmates after entry to prison. The aim of this study was to assess the changes in the consumption of psychoactive substance between the period before detention and during incarceration and to characterize the profiles of prisoners with similar consumption trajectories during incarceration.

**Methods and findings:**

A multicenter, cross-sectional study was performed in all of the prisons from one region of France. All prisoners incarcerated during their 3rd months, over 18 years old, and with a sufficient level of French fluency to participate in the study were recruited over a period of 12 months. A total of 800 prisoners were recruited. All prisoners were interviewed face-to-face by a trained interviewer. A majority of prisoners had used at least one psychoactive substance in the weeks prior to incarceration. During incarceration, a substantial reduction in alcohol and illicit drug consumption was observed. The initiation of consumption and an increase in consumption were primarily related to medications. Five different profiles of consumption before incarceration were identified. These profiles all had a high probability of migrating to a similar profile during detention, characterized by less severe consumption of psychoactive substances.

**Conclusions:**

Based on their consumption profile prior to incarceration, most prisoners would benefit from a specific medical evaluation as soon as possible following entry into detention. Prison could be an opportunity for reduced consumption and/or the initiation of treatment for the majority of prisoners, despite the pejorative development observed for a minority of prisoners during incarceration.

## Introduction

Despite bans, health professionals are regularly alarmed by the illicit substances or drugs that are diverted from their intended use in jail. Twenty years ago, an investigative report from the French Senate highlighted the elevated number of drugs users among incoming prisoners [1]. The same year, the RESSCOM survey described the use of psychoactive substances, illicit substances and drugs in prisons, including opioid maintenance treatments that were diverted from their intended use [2]. More recently, French studies reported a consumption prevalence for prisoners of between 10.8% and 83% of prisoners [3–11], which is similar to the prevalences reported in other international studies [12]. Others studies have focused on risk practices [2, 13–16] and highlighted injection practices inside prison with shared syringes. These consumption patterns are associated with important judicial and health consequences. Suicide ideation expressed by inmates upon entering custody appears to be correlated with addictive tendencies [17]. Moreover, loco-regional infectious complications (*e*.*g*., abscesses, wounds, and venous alterations) and general complications (*e*.*g*., lung disorders, HIV, HBV, and HCV), have been associated with risky consumption patterns [13–15, 18–20], especially for those in custody, where harm-reduction measures are limited. Finally, psychoactive substance consumption in custody appears to be linked with an increase in mortality upon release from prison [6, 21]; in particular, there is a major risk of overdose within two weeks of release from detention [22].

Whether some inmates continue to use or start using psychoactive substances in prison, entry into detention can be an opportunity for medical care and the cessation of substance use. Few studies have examined the consumption trajectories of inmates after entry to prison. However, many French government plans and expert reports have recently stressed the need for these studies. The knowledge gained from these studies would make it possible to adapt care policies for implementation in France and, in particular, to improve the identification of consumption and its management.

In France, the assessment of the abuse and dependence potential of psychoactive substances is performed by a specific monitoring system, called Addictovigilance [23–25], that is based on a network of 13 centers (French Addictovigilance Network "FAN") throughout France, located in university hospitals and associated with the ANSM (French national medicine agency). The primary missions of these centers are to collect and evaluate cases of abuse and dependence for psychoactive substances, to inform health professionals and to conduct research. FAN has already performed studies enabling a transversal approach to the consumption of psychoactive substances in detention [26], but the evolution of consumption between the beginning of detention and the months following incarceration has not been explored.

The aim of the consumption of substances and medicine drugs in prisoners (COSMOS) study was to assess the changes in psychoactive substance consumption patterns between the period before detention and the period during incarceration.

## Methods

### Study oversight

This regional prospective observational study was conducted by the Nantes Addictovigilance Center. The study was funded by a grant from the Health Regional Agency (ARS) and the Interministerial Mission for the Fight Against Drugs and Addictive Behaviours (Mission Interministérielle de la Lutte Contre les Drogues et les Conduites Addictives—MILDECA). It was monitored by a multidisciplinary steering committee composed of pharmacologists, psychiatrists who specialize in addiction, biostatisticians, and prison health professionals.

The COSMOS study was approved by the French Directorate of Prison Administration on September 16, 2013, and by an independent local ethics committee (Groupe Nantais d’Ethique dans le Domaine de la Santé - GNEDS) on October 21, 2014. The COSMOS study was conducted in accordance with the National Commission of Information Technology and Liberties (CNIL) rules regarding data management and analysis. The interviewer was in charge of providing clear information regarding the COSMOS study, and all prisoners provided oral statements of non-opposition to participation in the study in accordance with the Declaration of Helsinki.

### Patients

The study was proposed to every subject. To be eligible, prisoners had to be in one of the six prisons of the French Pays-de-la-Loire area (representing more than 3.5 million inhabitants), to be in their 3rd month of incarceration and to give consent.

All participants were prisoners who were over 18 years old (the age of majority in France) and who had a sufficient level of French fluency to participate in the study. Recruitment in the six prisons occurred between May 1, 2015, and April 30, 2016.

### Study procedures

Each inmate was assessed once completely anonymously via an *ad hoc* hetero-questionnaire built and validated by the steering committee.

Inmates were informed during the entry interview that they would be contacted during their incarceration to answer the questionnaire. During their 3rd month of incarceration, the prison staff sent a letter to the detainees suggesting a meeting to complete the study questionnaire. The detainees were free to accept or to refuse to go to the appointment. There was no obligation to attend the interview.

The completion of the questionnaire took place in a secure area of the prison by a person trained for the study who was completely independent of the prison. This person did not know the name of the prisoner to guarantee anonymity. There were no signs or annotations to identify the inmate who completed the questionnaire. The information leaflet was read to the subject, and the subject's oral statement of non-objection was collected prior to data collection. If after reading the information the subject did not wish to participate, he was free to terminate the interview. Non-participation in the study had no negative consequences for the subject. This procedure was approved by an independent ethics committee (study oversight section).

Once the questionnaire was completed, the evaluator anonymously and randomly filed it with the other questionnaires already collected. The stack of questionnaires was then sent to the Nantes Addictovigilance center for data entry and analysis.

The data included sociodemographic data (age, sex) and were divided into 3 parts:—Information regarding the consumption of psychoactive substances in the weeks preceding incarceration was the first set of data obtained from the detainee (alcohol, tobacco, illicit substances, prescribed drugs and medications consumed outside of a medical context), followed by the change in pre-existing consumption patterns during incarceration (cessation, reduction, increase, or no change).

- The initiation of psychoactive substance use during incarceration was the second question (alcohol, tobacco, illegal substances, prescribed drugs and drugs consumed outside of a medical context).

The following information was obtained for all substances and medications: frequency of consumption, duration of consumption, and route of administration. For medications, whether they were prescription drugs, whether they were used in accordance with the prescription and whether they were ingested in massive amounts were assessed during detention. The questionnaire included one open-ended question concerning the reason for drug consumption changes that were categorized before descriptive analysis (see S1 Table).

- Information pertaining to risky practices (nasal and/or injectable drug use) before and during incarceration was then obtained. When nasal and/or intravenous administration was used, prisoners were asked to supply information regarding the risky behaviors associated with the use of the nasal and/or intravenous administration route (*e*.*g*., the sterilization, sharing, and cleaning of materials).

### Outcomes

The primary objective was to assess the changes in psychoactive substance consumption patterns between the period before detention and the period during incarceration. The primary outcomes were changes in drug prevalence rates before and during incarceration. The secondary objective was to characterize the profiles of prisoners with similar consumption patterns and pattern changes in the period before incarceration and the period during incarceration. The secondary outcomes were the number of different profiles and transitions identified using a latent transition analysis.

### Statistical analysis

The characteristics of the population are described by the use of quantitative and qualitative variables. For normally distributed, quantitative continuous variables, the mean and standard deviation are used. For non-normally distributed, quantitative variables, the median and interquartile ranges are used. For categorical variables, the frequencies and percentages for each modality are displayed. The prevalence of consumption before and during incarceration was computed for each substance, with a 95% confidence interval. Relative differences and absolute differences were also computed, with 95% confidence intervals.

Latent transition analysis was used to examine changes in consumer profiles before incarceration and during incarceration. The PROC LTA developed by Collins et al. [27, 28] was used to build the model. In latent transition analysis, the profile of the inmate at a given time (in this study, before or during incarceration) constituted a "status". Several models were tested, with a number of statuses between 2 and 7. The choice of the best number of statuses was achieved by comparing the averages of the Bayesian Information Criterion (BIC) index values resulting from the construction of 1000 models. The number of statuses corresponding to the lowest average BIC was retained. To select the most suitable model for a given class number, the model with both the lowest BIC and the highest frequency of occurrence among the 1000 iterations was selected. In the case of similar BICs between two different numbers of statuses (that is, having overlapping 95% confidence intervals), the most appropriate models for each number of statuses were compared in terms of interpretability, and the most interpretable model was retained.

A second step was carried out to better characterize the profiles. Each patient was assigned the status for which the probability of belonging was highest. The sets of patients (one per identified status) were then cross-referenced and compared (age, frequency of use of substances, routes of administration) through ANOVA if the variable was continuous or a Chi^2^ test if the variable was categorical.

The final model is described by a set of parameters. The first set of parameters is a set of probabilities of a positive response to the item (here, the consumption of a substance). The second set of parameters is a set of probabilities of belonging to a given profile at each of the moments studied, comparable to a prevalence of the profile considered in the population studied at a given time. Finally, a third set of parameters was constituted, representing the probabilities of transition from a profile at a time t to another profile.

We used SAS 9.4 and proc LTA version 1.2.3.

## Results

### Study population

A total of 1,547 prisoners met the inclusion criteria. The number of subjects included per facility was proportional to the size of each facility; the sample is therefore representative of the population of prisons in our region.

Among them, 517 could not be interviewed because they were not available at the time of the questionnaire (at work, studying, receiving visitors or ill); thus, 1,030 were asked to participate in the study. A total of 226 prisoners refused to participate (21.9%) and 804 agreed to participate (78.1%). Four prisoners were excluded from the statistical analysis because they did not meet the inclusion criteria (incarceration for only 2 months). Finally, 800 questionnaires were retained in the statistical analysis. These subjects were mostly male (97.6%), and the mean age was 33 years (33.2, Standard Deviation (SD) = 11.3). The mean length of incarceration was 3.5 months (SD = 0.6).

### Changes in the prevalence of consumption

**Fig 1:**
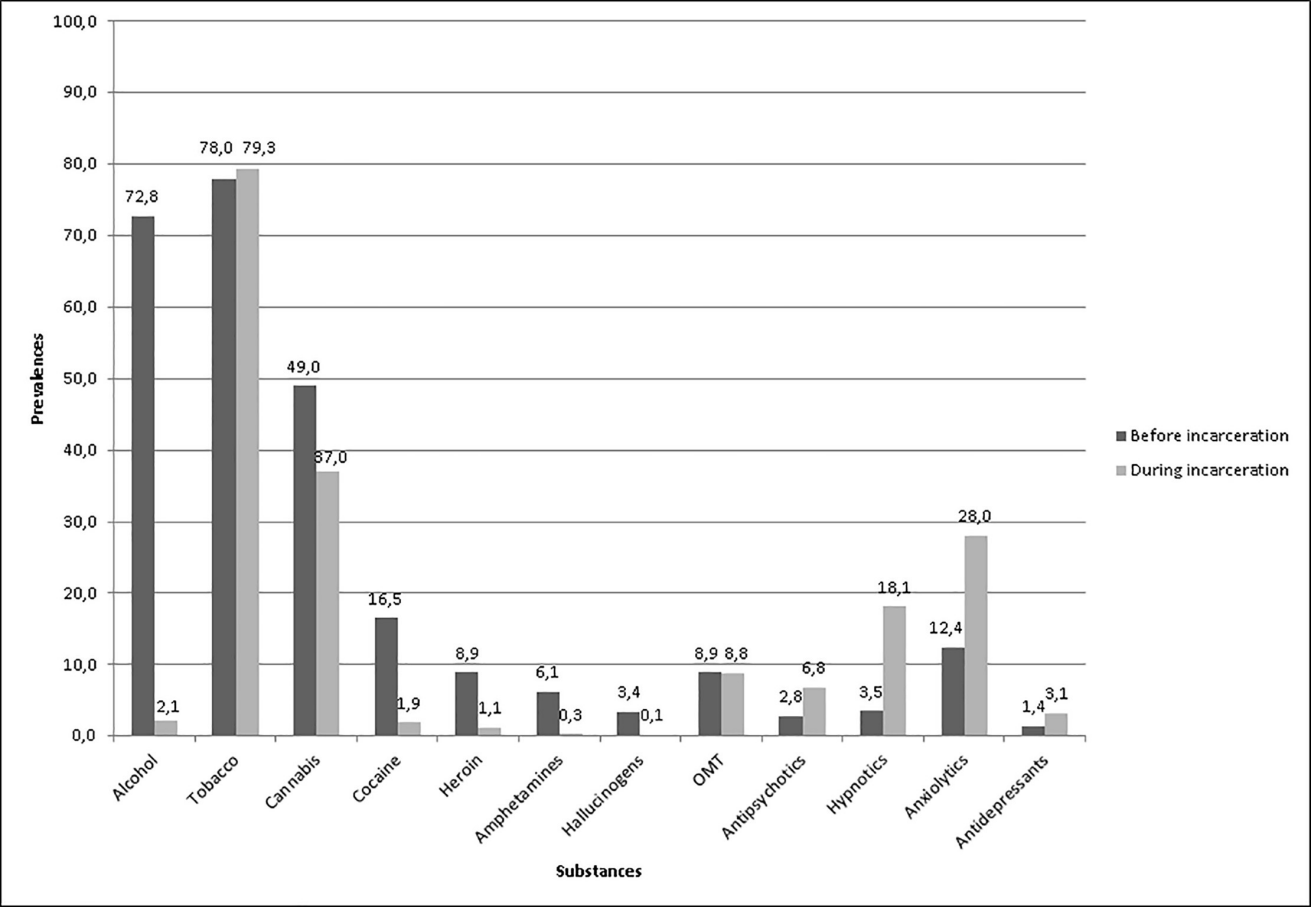
Changes in prevalence according to the study period. OMT, opiate maintenance treatment.

The results are displayed in Table 1 and Fig 1.

**Table 1:** Changes in the prevalence rates of substance and medicine consumption before and during incarceration.

Substance	Use before incarceration (N, %)	Consumptions characteristics before incarceration (%)	Use during incarceration (N, %)	Absolute variation (%,95% CI)	Relative variation (%)
Alcohol	582 (72.8%)	For several years (90.7%) Daily (36.6%) Weekly (27.5%) Casual (35.9%)	17 (2.1%)	-70.7 [-73.8 ; -67.5]	-97.1
Tobacco	624 (78.0%)	For several years (97.6%) Daily (99.2%) Casual (0.8%)	634 (79.3%)	1.3 [-0.05 ; 0.3]	+1.7
Cannabis	392 (49.0%)	For several years (95.7%) Daily (65.0%) Weekly (12.5%) Casual (22.2%)	296 (37.0%)	-12.0 [-14.0 ; -9.0]	-24.5
Cocaine	132 (16.5%)	For several years (75.0%) Daily (19.7%) Weekly (30.3%) Casual (50.0%)	15 (1.9%)	-14.5 [-17.1 ; -12.1]	-88.5
Heroin	71 (8.9%)	For several years (80.3%) Daily (49.3%) Weekly (15.5%) Casual (35.2%)	9 (1.1%)	-7.8 [-9.6 ; -5.8]	-87.6
Opiate Maintenance treatments	71 (8.9%) Prescribed (84.9%) Misused (24.6%)	For several years (71.2%) Daily (74.0%) Per-os (89.0%)	70 (8.8%) Prescribed (89.4%) Misused (12.3%)	-0.1 [-0.7 ; 1.3]	-1.1
Anxiolytics	102 (12.4%) Prescribed (90.2%) Misused (4.5%)	For several years (66.4%) Per-os (98.2%)	(28.0%) Prescribed (95.2%) Misused (19.7%)	15.6 [12.8 ; 18.5]	+125.8
Hypnotics	29 (3.5%) Prescribed (86.7%) Misused (16.7%)	For several years (76.7%) Per-os (86.7%)	145 (18.1%) Prescribed (97.4%) Misused (20.8%)	14.6 [11.9 ; 17.0]	+417.1

Regular consumption corresponds to at least once per week, casual corresponds to less than weekly. %, percentage; CI, Confidence Interval

Before incarceration, only 56 (7%) had not used any psychoactive substances in the weeks prior to incarceration. The most consumed substances were alcohol, tobacco and cannabis, characterized as consumption for years on a daily/weekly basis. The reported medications were largely prescribed. Prisoners who reported the daily consumption of psychoactive substances (excluding tobacco, which is available in jail) accounted for 471 (58.9%) of subjects. Prisoners had used approximately 3 different psychoactive substances in the weeks prior to incarceration (2.75, SD = 1.9, maximum 17).

During incarceration, 99 prisoners (12.4%) had not used any psychoactive substances (due to the cessation of psychoactive substance consumption in the weeks prior to incarceration and/or the lack of initiation of any psychoactive substance consumption during incarceration). A total of 249 prisoners (31.1%) reported the daily use of psychoactive substances. Prisoners used one to two psychoactive substances during their incarceration (2.0, SD = 1.5, maximum 9). As shown in Table 1, the highest reduction in prevalence was observed for alcohol use followed by illicit drugs. The highest increase in prevalence was observed for prescribed medications, and the massive intake of medications in prison was reported by approximately 15% of prisoners. The major trend was a global increase in the consumption of prescribed medications and a decrease in the consumption of other substances with the exception of tobacco and opiate maintenance treatments; nevertheless, within these important trends, for some subjects, psychoactive substance consumption remained unchanged during incarceration. This was the case for (i) some medications when consumption was reported as day-to-day before incarceration and (ii) for other illicit substances and alcohol when consumption was reported as occasional before incarceration.

Globally, the reasons for change varied according to the frequency of use before incarceration for each substance (S1 Table). As a rule, for consumers who stopped or decreased their consumption, the reasons associated with personal decisions or medical care were more often reported in daily users (p<0.05 for alcohol, tobacco, cannabis, cocaine, and heroin), and changes in the habitual context of consumption were more often reported by occasional users (p<0.05 for alcohol, cannabis, cocaine, and heroin). The difficulty in obtaining supplies (either the substance or the equipment required for administration) was often reported by occasional and daily users. For consumers who increased or initiated consumption, seeking positive sensations, avoiding withdrawal or coping with boredom were the most commonly reported reasons (see details S1 Table).

The following psychoactive substance initiation was reported by 311 prisoners: 83% prescribed medications, 6% tobacco, 6% cannabis, 4% medications obtained without prescription and 1% cocaine and heroin.

A total of 149 prisoners used nasal or intravenous administration before incarceration. Regarding risky behaviours in prison, 29 prisoners used nasal administration and 1 prisoner used intravenous administration during incarceration. For nasal administration in prison, sterile equipment was used for approximately half of the prisoners, and when equipment was not sterile, it was rarely disinfected before consumption. Most of the nasal users did not share equipment.

### Number of different profiles and transitions identified using a latent transition analysis

The latent transition model that best fitted our data depicted five different profiles before and during incarceration.

#### Profiles of consumers in the weeks prior to incarceration (Table 2)

**Table 2:** Probabilities of positive items (consumptions) and probabilities of belonging to a given status for each status (profile of consumers) before incarceration.

	Profile 1 17,2%	Profile 2 33,4%	Profile 3 8,9%	Profile 4 27,2%	Profile 5 13,3%
**Mean Age (SD)**	40.0 (15.0)	32.7 (11.1)	38.1 (9.7)	28.2 (7.4)	32.3 (7.9)
**Alcohol**	0.53	0.71	0.80	0.82	0.78
**Tobacco**	0.00	0.96	0.96	0.91	0.96
**Cannabis**	0.02	0.27	0.38	0.98	0.72
**Cocaine**	0.01	0.00	0.12	0.19	0.75
**Heroin**	0.00	0.00	0.00	0.03	0.61
**Amphetamines**	0.00	0.00	0.03	0.05	0.34
**Hallucinogens**	0.00	0.00	0.00	0.00	0.18
**Opiate** **Maintenance Treatment**	0.00	0.00	0.06	0.03	0.62
**Antipsychotics**	0.00	0.01	0.21	0.00	0.04
**Antidepressants**	0.02	0.00	0.11	0.00	0.00
**Hypnotics**	0.02	0.00	0.22	0.00	0.09
**Anxiolytics**	0.11	0.00	0.78	0.03	0.22

Profile 1 corresponds to a profile of prisoners who are older than those in the other profiles and who most often present only a high probability of alcohol consumption. The frequency of alcohol consumption varied from occasional (39.7%) to daily (35.6%).

Profile 2 was the most prevalent and corresponded with middle-aged prisoners who had a high probability of alcohol and tobacco used and were associated with moderate cannabis use. However, fairly frequent daily cannabis use (43.0%) was found, while alcohol consumption was occasional in 46.2% of cases.

Profile 3 differed from all of the other profiles because of its higher psychotropic drug consumption probability (excluding OMT). For this profile, daily alcohol consumption was higher (64.1%) than for all of the other profiles, and cannabis use was occasional (52.0%). Cocaine use was more frequently occasional (66.7%).

Profile 4 included a large proportion of prisoners who were significantly younger than those in the other profiles (28.2 years, SD = 7.4 years). This profile was characterized by a very high probability of alcohol, tobacco and cannabis consumption. For this profile, daily cannabis users were more common (75.1%) than for all of the other profiles. Alcohol consumption was more often occasional or regular (68.0%), and cocaine use was occasional (68.3%).

Profile 5 was distinguished from the other profiles because of its much higher probability of consumption for illicit substances and because of the consumption of OMT. This profile appears to be more severe than the other profiles for all consumption, with daily consumption of alcohol and cannabis reported by 57.3% and 65.4% of prisoners, respectively. Moreover, this profile is the only profile that contains prisoners using intravenous administration. Cocaine use was more often regular (38.3%), and heroin use was often daily (49.2%).

#### Profiles of consumers during the first months of incarceration (Table 3)

**Table 3:** Probabilities of positive items (consumptions) and probabilities of belonging to a given status for each status (profile of consumers) during incarceration.

	Profile 1 17,5%	Profile 2 31,8%	Profile 3 14,6%	Profile 4 23,2%	Profile 5 12,9%
**Alcohol**	0.00	0.02	0.01	0.03	0.04
**Tobacco**	0.00	0.97	0.97	0.96	0.94
**Cannabis**	0.02	0.05	0.29	1.00	0.60
**Cocaine**	0.00	0.00	0.00	0.02	0.12
**Heroin**	0.00	0.00	0.00	0.00	0.09
**Amphetamines**	0.00	0.00	0.00	0.00	0.01
**Hallucinogens**	0.00	0.00	0.00	0.00	0.00
**Opiate** **Maintenance Treatment**	0.00	0.00	0.00	0.00	0.68
**Antipsychotics**	0.01	0.00	0.27	0.04	0.13
**Antidepressants**	0.04	0.00	0.13	0.00	0.04
**Hypnotics**	0.11	0.07	0.43	0.12	0.38
**Anxiolytics**	0.18	0.09	0.78	0.12	0.61

%, percentage

Profile 1 described prisoners who used virtually no substances.

Profile 2 was the most prevalent and, described prisoners who presented a high probability of heavy tobacco use only.

Profile 3 was primarily characterized by the high probability of anxiolytic, hypnotic, antipsychotic and antidepressant use.

Profile 4 was similar to profile 2, except for the very high probability of cannabis consumption.

Profile 5 differed primarily from the other profiles by the high probability of OMT use, which was almost null in the other profiles. This was the only profile for which current illicit substance use (excluding cannabis) was still associated with a non-negligible probability.

#### Transition between profiles before and during incarceration (Fig 2)

**Fig 2:**
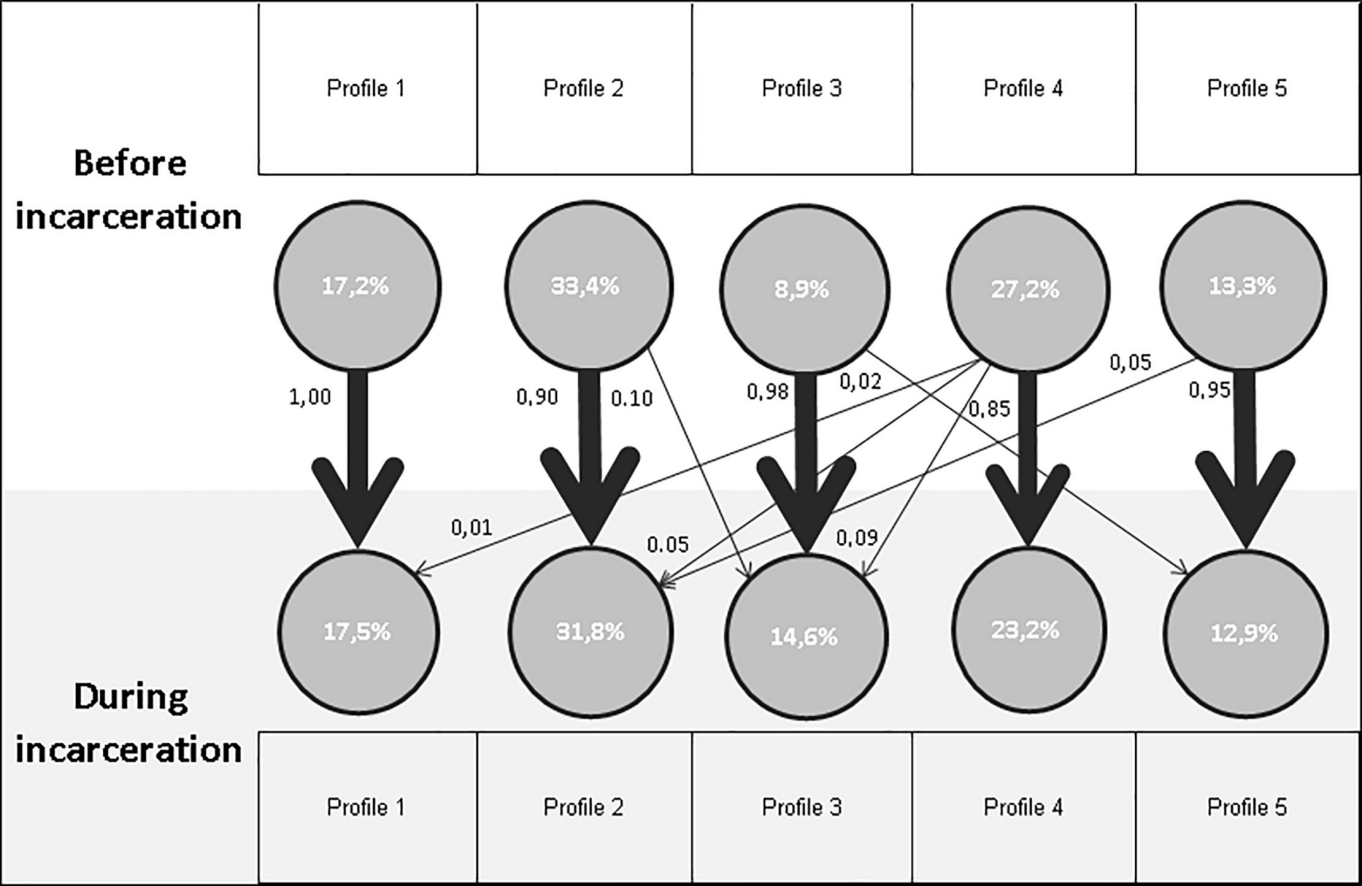
Transition between profiles before and during incarceration. %, percentage.

Overall, all of the prisoners from a single profile before incarceration have high probabilities of migrating to a similar profile during detention.

Prisoners from profile 1 had a high probability of stopping alcohol consumption, without initiating other psychoactive substance consumption (except anxiolytics).

Prisoners from profile 2 had a high probability of stopping alcohol and cannabis consumption. A small proportion of these prisoners had a probability of continuing cannabis consumption and/or initiating psychotropic medicine consumption.

Prisoners from profile 3 had a high probability of continuing their consumption (except for alcohol). A small proportion of these prisoners migrated to final profile 5, these prisoners had a probability of initiating an OMT. Prisoners from profile 4 had a high probability of stopping alcohol and cocaine and of continuing and perhaps initiating cannabis consumption. A small proportion had a probability of also stopping cannabis and tobacco consumption (profile 1), of stopping only cannabis consumption (profile 2) or of initiating medicine consumption (profile 3).

Prisoners from profile 5 had a high probability of stopping illicit consumption (except cannabis). OMT had a high probability of being retained and even initiated, and psychotropic medications had a high probability of being initiated. A small proportion had a probability of stopping all consumption (except tobacco), without the initiation of new psychotropic medications (profile 2).

## Discussion

### Comparison with other studies–before incarceration

It was first established that there is a significant prevalence of daily psychoactive substance consumption (excluding tobacco) among prisoners before incarceration (58.9%), primarily alcohol and cannabis. Cannabis is the most common illicit substance consumed (49%), far more prevalent than cocaine (16%) and heroin (8.8%). These prevalence rates are much higher than those observed for the French general population (cannabis 11%, cocaine 1.1% and heroin 0.2%) [29]. This illustrates the phenomenon of the concentration of illicit substance users in prison, which could be related in particular to the high frequency of incarcerations for drug legislation infractions (15% of incarcerations in 2012) [30, 31]. Other factors may also be involved, such as a lower socio-economic status or more frequent psychiatric disorders in the population of people that are likely to be incarcerated [4, 32]. We can assume that our prisoner population was representative of all prisoners, as previous studies in France with more prisoners showed similar prevalence rates for psychoactive substance consumption before incarceration [3, 5, 9]. Other countries also reported high prevalence rates for substance consumption before incarceration [33–35]. A recent systematic review of studies with ICD or DSM diagnoses found a prevalence of 24% for alcohol use disorders and a prevalence of 30% for drug use disorders before incarceration [36]. Our prevalence are more than twice as high as what this review report, but we determine consumption prevalence but no substance use disorder. The authors found evidence of increasing drug use disorders for prisoners entering prison over the past 3 decades. The authors recommended the identification of individuals with high treatment needs and the increased availability of detoxification management at entry (in particular for alcohol and opioids).

### Comparison with other studies–during incarceration

Regarding consumption during incarceration, we found 31.1% of prisoners reported daily consumption of psychoactive substances. Studies assessing consumption in French prisons using self-assessments found higher prevalence rates (cannabis 49% [10]; tobacco 75% and cannabis 37% [11]). When assessing consumption with interviews, prevalence rates are lower (15% dependence (except alcohol) [4]; 27.9% abuse and dependence for illicit drugs and misused medication [6]). International literature regarding consumption during detention is scarce, perhaps due to the difficulty of asking prisoners to report their actual consumption in a context where consumption is prohibited. We found a prevalence of 53.8% for any substance use in Mexico City [37] and 59.9% in Spain [38].

### Risks identified

Before incarceration, 471 prisoners (58.9%) reporting the daily consumption of psychoactive substances or prescription drugs, either with or without prescriptions. All of these prisoners, because of their consumption habits, would benefit from a minimal medical evaluation as soon as possible and follow-up upon entry into detention, considering the risk of withdrawal syndrome and to ensure the continuity of care for prisoners receiving medical treatment. These results are in accordance with those reported by Fazel et al. [36].

Another worrying result is the high prevalence of the massive ingestion of medications (15%) during detention. The reasons most often used to justify these incidents are "forgetting", "hovering", "no longer thinking about everyday life", and "escaping". Lethality has never been mentioned in our interviews, but the line between the desire to forget and escape and the desire to commit a life-threatening act is tenuous. This is a very important consequence, especially because France has one of the highest suicide rates for incarcerated individuals in the world [36].

The use of the nasal route was largely more often reported than the use of the intravenous route, both before and during incarceration. The majority use of the nasal route during incarceration has already been documented and is partly explained by the scarcity of syringes in prisons [2]. However, it should be noted that our study focuses on a limited period after incarceration, and the use of the intravenous route may occur with longer detention. Two studies in France showed the prevalence of injection experiences in prison to be approximately 15% when drug users with previous incarcerations were interviewed [20, 32]. More worrying, 30% of these prisoners reported having shared equipment for intravenous administration in prison [20]. In our study, it appears that, during incarceration, nasal-associated practices involved less use of sterile equipment and less disinfection prior to use, which increases the risk of contamination. Previous studies also highlighted the need for the development of risk reduction programs in prisons, but most of them focused on the risks associated with intravenous administration [39–42]. Studies regarding nasal administration risks in prison are lacking, and we must encourage the development of such studies.

### Prison: A chance?

Even if the poor evolution of psychoactive substance consumptions habits is observed for some prisoners, our results show a quantitative decrease in consumption and a qualitative change in consumption, with some of the prisoners scheduling a medical follow-up. Thus, in our study, there was a significant decrease in the prevalence of alcohol and all illicit substance consumption during incarceration, with all consumption (except cannabis) decreasing by more than 85%. The prevalence of cannabis consumption decreased to a lesser extent. The reasons given for stopping substance use were primarily personal decisions, the difficulty of obtaining substances and changes in the context of consumption (*e*.*g*., the disappearance of the festive context). The decrease in alcohol and illicit substance consumption was accompanied by an increase in prescriptions for anxiolytics, hypnotics and neuroleptics (primarily cyamemazine), which were initiated during incarceration. It is important to highlight that the latent transition model reinforces these results. Very few prisoners from profiles without multidrug use before detention evolved towards a multidrug use profile in the first three months of detention. During incarceration, most of the prisoners evolved to a profile with less severe consumption characteristics, with significant decreases in alcohol and illicit substance consumption (except cannabis) and increases in psychotropic medicine use probabilities. The increase in medicine use could be due to medical management of the acclimatization to the prison environment and/or the management of withdrawal symptoms related to the cessation of alcohol and illicit drug consumption. Sleep disorders are common in prison and can be explained by the conditions of imprisonment and the over-occupation of prisons [43]. Regarding OMT, no significant change in prevalence rates during incarceration was observed. Most consumption rates were retained or increased when prisoners reported daily use before incarceration. This result appears to be compatible with the rational use of these medications in the context of opioid withdrawal that began before entering prison. Regarding other psychotropic medications, we found little reported use of antidepressants and antipsychotics during incarceration. Falissard et al. [4] showed higher prevalence rates of schizophrenia and mood disorders in French prisoners. The prisoners we interviewed were at the beginning of their incarceration and may not yet have been seen by a doctor for their disorders or the treatment may not yet have been initiated. We can assume that, at the beginning of incarceration, the detention environment can have repercussions on mood, and, as a consequence, make the diagnosis of mood disorders more challenging. If diagnoses of mood disorders are delayed, so are the antidepressant prescriptions. The increase in anxiolytic and hypnotic prescriptions in the early detention period confirmed this hypothesis; medicine prescriptions are symptomatic of the experiencing detention for the first time.

### Strengths and limits of the study

This work is characterized by several remarkable elements. First, the number of prisoners recruited, which surpassed the number of prisoners originally planned, allowed us to update completely and from various angles the current knowledge regarding psychoactive substance consumption in prison. Previous studies showed differences in prevalence rate, which may be related to the use of different methodologies and, in particular, the length of detention can lead to a memory bias regarding the assessment of consumption before incarceration; the use of a self-questionnaire versus an interview can also lead to differences, as we know the rate of illiteracy in prison. From this point of view, our study has the following strengths: questionnaires were administered face-to-face after a relatively short time of incarceration (during the third month); the questionnaire evaluated consumption before incarceration; and the questionnaire simultaneously evaluated changes in consumption during the first three months, when the subject is adapting to his new environment. In addition, strict anonymity was respected; therefore, we can assume a certain reliability of the answers. However, if a bias exists, it may be an underestimate of consumption and the consumption practices within each prison. Finally, this is the first study that implements a latent transition approach to identify patterns of substance use in prison and their evolutions during the first months of incarceration. This approach has allowed us to illustrate the phenomenon of substance use in a global way and to highlight certain otherwise invisible mechanisms. To our knowledge, this is the first time that the consumption trajectories of prisoners have been assessed.

It should be noted, however, that latent transition analysis is essentially descriptive, and these results must be considered to be hypotheses tested in other subsequent works. These results relate to the first three months of incarceration, and other phenomena may occur later in the life of the prisoner. In addition, it should be considered that it is possible that a phenomenon of underreporting of consumption exists. Indeed, the consumption of substances in prison remains challenging for the prisoners, as it could constitute a weakness for prisoners and could potentially reinforce the difficulties encountered daily [2]. The prisoners in our study were mostly male, therefore, even if similar results were observed in a female prison population [44], the results cannot be generalized to all prisoners. Finally, this study was conducted in Pays-de-la-Loire and the generalization of these results to other regions is subject to caution.

## Conclusion

In conclusion, our study highlighted a clear decrease in psychoactive substance consumption (excluding tobacco) for a large majority of prisoners. There was also an improvement in the good use of medicine consumption. Thus, there is a feeling that prison could constitute an opportunity for reduced consumption and/or the initiation of treatment for the majority of prisoners, despite the increase in harmful consumption patterns observed for a minority of prisoners during incarceration.

## Supporting information

S1 Table Three most frequently reported reasons for change by substance, according to the type of change. Legend: %, percentage.
(DOCX)
