# Supplementary material for: Consumption of psychoactive substances in prison: Between initiation and improvement, what trajectories occur after incarceration? COSMOS study data
Source: PLoS One. 2019 Dec 4;14(12):e0225189. doi: 10.1371/journal.pone.0225189 (PMC6892542; doi:10.1371/journal.pone.0225189)
Supplement: S1 Table — Legend: %, percentage. (DOCX) [file pone.0225189.s001.docx]

| **Type of change** | **Stop** | | **Diminution** | | **Increase** | | **Initiation** |
| --- | --- | --- | --- | --- | --- | --- | --- |
| **Frequency before** | **Daily** | **Other** | **Daily** | **Other** | **Daily** | **Other** | **/** |
| Alcohol | **N=205**  Cannot access anymore (69.8%)  Medical care (24.9%)  Personal decision (21.5%) | **N=360**  Out of context (52.2%)  Cannot access anymore (51.7%)  Personal decision (11.1%) | **N=16**  Cannot access anymore (56.3%)  Out of context (25.0%)  Personal decision (18.8%) | | **N=0**  / | | **N=0**  / |
| Tobacco | **N=18**  Personal decision (38.9%)  Cannot access anymore (16.7%)  High cost (11.1%) | / | **N=184**  Personal decision (35.9%)  High cost (21.7%)  Change in pace (16.3%) | / | **N=228**  Coping with boredom (77.2%)  Seeking positive sensations (35.5%)  Replacing another product (7.5%) | / | **N=31**  Replacing another product (51.6%)  Seeking positive sensations (25.8%)  Coping with boredom (19.4%) |
| Cannabis | **N=60**  Personal decision (58.3%)  Cannot access anymore (28.3%)  Out of context (19.7%) | **N=62**  Personal decision (50.0%)  Out of context (30.6%)  Cannot access anymore (30.6%) | **N=132**  Cannot access anymore (54.5%)  Personal decision (28.8%)  High cost (28.0%) | **N=37**  Cannot access anymore (29.8%)  Personal decision (27.6%)  Out of context (29.8%) | **N=22**  Seeking positive sensations (59.1%)  Coping with boredom (50.0%)  Stop thinking, avoiding withdrawal (13.6%) | **N=15**  Seeking positive sensations (53.3%)  Coping with boredom (53.3%)  Stop thinking, , avoiding withdrawal (40.0%) | **N=28**  Seeking positive sensations (53.6%)  Replacing another product (28.6%)  Stop thinking, avoiding withdrawal (25.0%) |
| Cocaine | **N=24**  Cannot access anymore (50.0%)  Personal decision (37.5%)  Medical care (25.0%) | **N=96**  Personal decision (46.9 %)  Out of context (36.5%)  Cannot access anymore (26.0%) | **N=9**  Cannot access anymore (44.4%)  High cost (44.4%)  Out of context (33.3%) | | **N=0**  / | | **N=3**  / |
| Heroin | **N=31**  Personal decision (41.9%)  Cannot access anymore (25.8%)  Out of context (29.0%) | **N=32**  Medical care (56.3%)  Personal decision (37.5%)  Cannot access anymore (18.8%) | **N=5**  / | | **N=0**  / | | **N=2**  / |
| Opiates Maintenance treatment | **N=14**  Personal decision (28.6%)  Cannot access anymore (21.4%)  Out of context (14.3%) | | **N=19**  Personal decision (42.1%)  Cannot access anymore (15.8%)  High cost (5.3%) | | **N=15**  Stop thinking, avoiding withdrawal (13.3%)  Medical care (13.3%)  Seeking positive sensations (6.7%) | | **N=17**  Stop thinking, avoiding withdrawal (29.5%)  Medical care (5.9%)  Seeking positive sensations (5.9%) |
| Anxiolytics | **N=33**  Personal decision (18.2%)  Cannot access anymore (9.1%)  Intolerance (6.1%) | | **N=16**  Personal decision (25.0%)  Change of pace (6.2%)  Seeking positive sensations (6.2%) | | **N=18**  Stop thinking, avoiding withdrawal (16.7%)  Personal decision (11.1%)  Seeking positive sensations (5.6%) | | **N=173**  Stop thinking, avoiding withdrawal (5,2%)  Medical care (2.9%)  Seeking positive sensations (1.7%) |
| Hypnotics | **N=8**  / | | **N=3**  / | | **N=2**  / | | **N=132**  Seeking positive sensations (3.0%)  Replacing another product (1.5%)  Medical care (0.7%) |
